# The periscope sign as a new dermatoscopy finding to facilitate the diagnosis of furuncular myiasis

**DOI:** 10.1093/jtm/taae070

**Published:** 2024-05-16

**Authors:** Matiar Madanchi, Tamara Merkel, Hazem A Juratli, Riccardo Curatolo

**Affiliations:** Department of Dermatology, University Hospital of Basel, Burgfelderstrasse 101, 4055 Basel, Switzerland; Department of Dermatology, University Hospital of Basel, Burgfelderstrasse 101, 4055 Basel, Switzerland; Department of Dermatology, University Hospital of Basel, Burgfelderstrasse 101, 4055 Basel, Switzerland; Department of Dermatology, Dermatopathology Training Center, University Hospital Basel, Schönbeinstrasse 40, 4031 Basel, Switzerland; Institute of Medical Genetics and Pathology, University Hospital Basel, Schönbeinstrasse 40, 4031 Basel, Switzerland; Department of Dermatology, University Hospital of Basel, Burgfelderstrasse 101, 4055 Basel, Switzerland

## Abstract

In this text, we introduce the ‘periscope sign’ as a novel dermatoscopic finding in furuncular myiasis. This clinical sign may aid in diagnosing this rare condition, particularly in patients with a history of travel to endemic areas.

A 65-year-old woman presented to our emergency consultation with painful nodules on her back. The patient reported that she returned to Switzerland about 5 days ago after travelling to Congo. While in Congo, she experienced several mosquito bites that initially caused intense itching, however, not on her back where the lesions were located, but rather on her legs.

Upon clinical examination, we observed multiple painful furunculoid nodules with central crateriform ulceration, resembling a volcano (Figure 1A), especially in a lesion where purulent discharge was also observed (Figure 1B). Dermoscopically, we also noted erythematous lesions with a central larval breathing hole, resembling a periscope (Figure 2A–B). Therefore, we would like to suggest the ‘periscope sign’ as a possible new dermatoscopic sign for easily identifying this condition.

**Figure 1:**
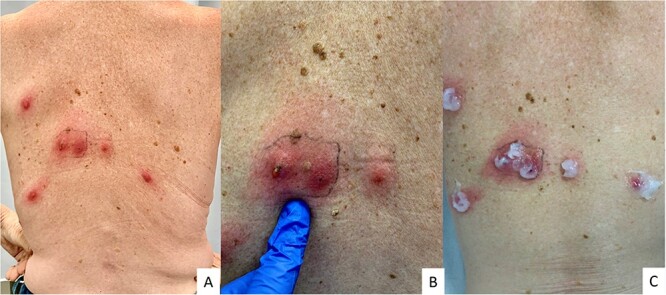
Clinical presentation of myiasis; multiple painful furunculoid nodules with central crateriform ulceration (A), purulent discharge from a lesion (B), application of pure Vaseline on the lesions (C)

**Figure 2:**
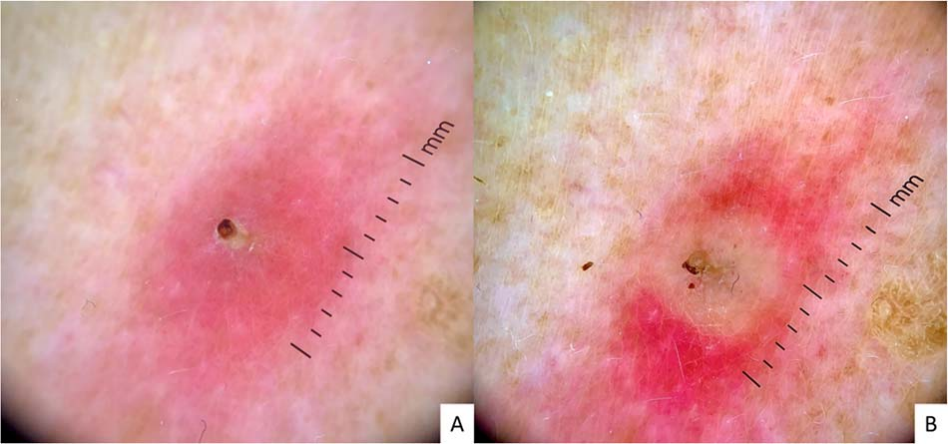
Dermatoscopic images; erythematous lesions with a central larval breathing hole, resembling a periscope (A and B)

In this case, we proceeded with the application of pure vaseline to occlude larval breathing (Figure 1C) and subsequently removed a total of eight larvae of varying sizes (Figure 3A). We then observed the larvae under a microscope at 10× magnification and could observe the larval breathing hole (Figure 3B, black arrow) and the presence of microspicules within the structure, allowing for a firm and stable anchorage in the subcutaneous tissue (Figure 3B, red arrow). This may explain the difficulty in removing these larvae, as they are well anchored in the subcutaneous tissue due to these spicules. The entomologist’s assessment identified the larvae as *Cordylobia anthropophaga*.

**Figure 3:**
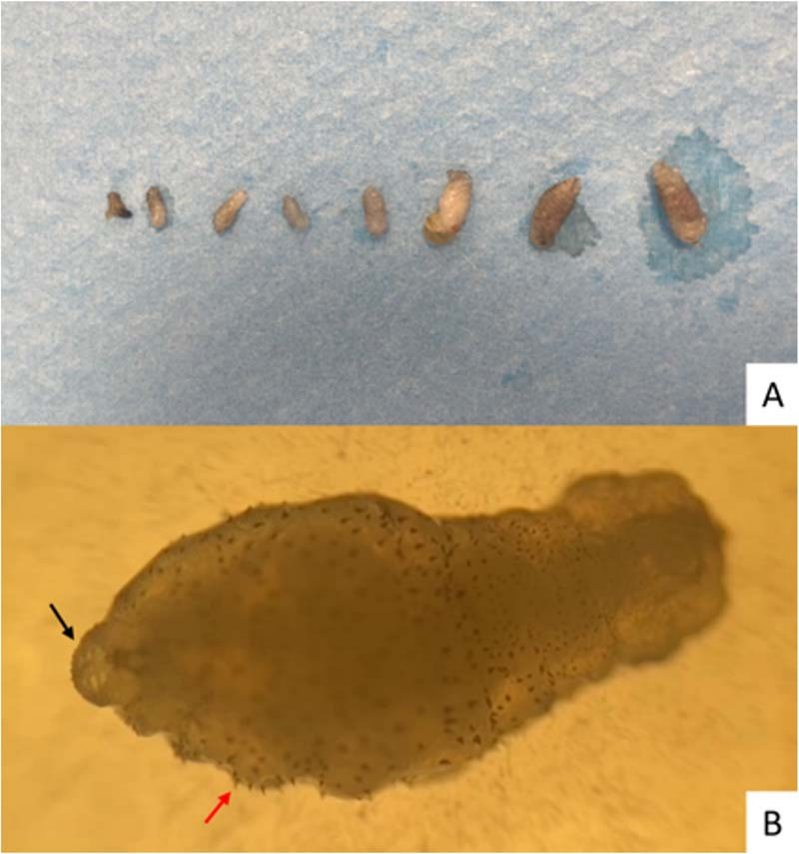
Macroscopic view of the eight removed larvae (A); microscopic finding at 10× magnification of the larva, showing the breathing hole (B) and the presence of microspicules within the structure (B)

Furunculoid myiasis is a form of dermatomyiasis in which fly larvae penetrate the skin and subcutaneous tissue, causing painful, suppurating nodules.^1^^,^^2^ Various fly species can cause furuncular myiasis, such as *Dermatobia hominis* in South America, *C. anthropophaga* in Africa, *Cuterebra* species in North America and *Wohlfahrtia* species in Europe or Pakistan.^3^^,^^4^

Lesions from *C. anthropophaga* are multiple, especially in areas covered by clothing. In fact, *C. anthropophaga* often deposits eggs on the ground or as more often seen in infections on humans, the eggs are deposited on clothes. Usually, in endemic areas, to prevent infections, it is necessary to iron clothes to prevent infection; however, if this is not done, the eggs can hatch, and the larvae, upon contact with the skin, can penetrate the subcutaneous tissue.^3^^,^^5^

In Switzerland, furuncular myiasis is rare and is often seen in patients returning from travels to these regions or in migrants originating from these regions of the world.

Therefore, we observed this particularity dermoscopically, which we have nicknamed the ‘periscope sign’. A periscope is an optical device that allows an observer to explore the entire horizon from a position where direct visibility is not possible, often used in submarines. This could be a fitting comparison, as the larva is located subcutaneously like a submarine, but to survive, it must expose part of its breathing hole to breathe. Furuncular myiasis is often a clinical diagnosis; however, a dermatoscope could be used to evaluate the lesions more closely. This could be a simple and easy-to-remember dermatoscope sign that would enable rapid recognition of this condition and appropriate therapeutic intervention.

## References

[1] Caissie R , Beaulieu F, Giroux M, Berthod F, Landry PÉ. Cutaneous myiasis: diagnosis, treatment, and prevention. J Oral Maxillofac Surg 2008; 66:560–8. 18280395 10.1016/j.joms.2007.09.005

[2] Weekes M , Matheson N, Coggle S, Gkrania-Klotsas E. Furuncular myiasis. BMJ Case Rep 2009; 2009:bcr0620092026. 10.1136/bcr.06.2009.2026 PMC3029061 21866234

[3] Francesconi F , Lupi O. Myiasis. Clin Microbiol Rev 2012; 25:79–105. 22232372 10.1128/CMR.00010-11 PMC3255963

[4] Nassar A , Abualiat A, El-Attar Y et al. A dermoscopic study of cutaneous myiasis: other findings. Int J Dermatol 2021; 60:840–3. 33682922 10.1111/ijd.15503

[5] Robbins K , Khachemoune A. Cutaneous myiasis: a review of the common types of myiasis. Int J Dermatol 2010; 49:1092–8. 20883399 10.1111/j.1365-4632.2010.04577.x

